# Family-based cognitive behavioural therapy versus family-based relaxation therapy for obsessive-compulsive disorder in children and adolescents (the TECTO trial): a statistical analysis plan for the randomised clinical trial

**DOI:** 10.1186/s13063-022-06799-4

**Published:** 2022-10-06

**Authors:** Markus Harboe Olsen, Julie Hagstrøm, Nicole Nadine Lønfeldt, Camilla Uhre, Valdemar Uhre, Linea Pretzmann, Sofie Heidenheim Christensen, Christine Thoustrup, Nicoline Løcke Jepsen Korsbjerg, Anna-Rosa Cecilie Mora-Jensen, Melanie Ritter, Janus Engstrøm, Jane Lindschou, Hartwig Roman Siebner, Frank Verhulst, Pia Jeppesen, Jens Richardt Møllegaard Jepsen, Signe Vangkilde, Per Hove Thomsen, Katja Hybel, Line Katrine Harder Clemmesen, Christian Gluud, Kerstin Jessica Plessen, Anne Katrine Pagsberg, Janus Christian Jakobsen

**Affiliations:** 1 Copenhagen Trial Unit, Centre for Clinical Intervention Research, Copenhagen University Hospital – Rigshospitalet, Copenhagen, Denmark; 2 Department of Neuroanaesthesiology, The Neuroscience Centre, Copenhagen University Hospital – Rigshospitalet, Copenhagen, Denmark; 3 Child and Adolescent Mental Health Center, Copenhagen University Hospital, Mental Health Services CPH, Copenhagen, Denmark; 4 Department of Clinical Medicine, Faculty of Health and Medical Sciences, University of Copenhagen, Copenhagen, Denmark; 5 Danish Research Centre for Magnetic Resonance, Centre for Functional and Diagnostic Imaging and Research, Copenhagen University Hospital − Amager and Hvidovre, Copenhagen, Denmark; 6 Department of Neurology, Copenhagen University Hospital − Bispebjerg and Frederiksberg, Copenhagen, Denmark; 7 Child and Adolescent Psychiatric Department, Region Zealand, Psychiatry, Research Unit, Roskilde, Denmark; 8 Center for Clinical Intervention and Neuropsychiatric Schizophrenia Research (CINS), Mental Health Center Glostrup, Copenhagen University Hospital – Rigshospitalet, Copenhagen, Denmark; 9 Department of Psychology, Faculty Social Sciences, University of Copenhagen, Copenhagen, Denmark; 10 Department of Child and Adolescent Psychiatry, Aarhus University Hospital, Aarhus, Denmark; 11 Applied Mathematics and Computer Science, Technical University of Denmark, Kgs Lyngby, Denmark; 12 Institute of Regional Health Research, The Faculty of Health Sciences, University of Southern Denmark, Odense, Denmark; 13 Division of Child and Adolescent Psychiatry, Department of Psychiatry, Lausanne University Hospital (CHUV) and University of Lausanne, Lausanne, Switzerland

**Keywords:** Obsessive-compulsive disorder, Cognitive behavioural therapy, Family-based psychoeducation/relaxation training, Randomised clinical trial, Statistical analysis plan

## Abstract

**Background:**

Obsessive-compulsive disorder (OCD) is a debilitating psychiatric disorder which affects up to 3% of children and adolescents. OCD in children and adolescents is generally treated with cognitive behavioural therapy (CBT), which, in more severely affected patients, can be combined with antidepressant medication. The TECTO trial aims to compare the benefits and harms of family-based CBT (FCBT) versus family-based psychoeducation/relaxation training (FPRT) in children and adolescents aged 8 to 17 years. This statistical analysis plan outlines the planned statistical analyses for the TECTO trial.

**Methods:**

The TECTO trial is an investigator-initiated, independently funded, single-centre, parallel-group, superiority randomised clinical trial. Both groups undergo 14 sessions of 75 min each during a period of 16 weeks with either FCBT or FPRT depending on the allocation. Participants are randomised stratified by age and baseline Children’s Yale–Brown Obsessive-Compulsive Scale (CY-BOCS) score. The primary outcome is the CY-BOCS score. Secondary outcomes are health-related quality of life assessed using KIDSCREEN-10 and adverse events assessed by the Negative Effects Questionnaire (NEQ). Primary and secondary outcomes are assessed at the end of the intervention. Continuous outcomes will be analysed using linear regression adjusted for the stratification variables and baseline value of the continuous outcome. Dichotomous outcomes will be analysed using logistic regression adjusted for the stratification variables. The statistical analyses will be carried out by two independent blinded statisticians.

**Discussion:**

This statistical analysis plan includes a detailed predefined description of how data will be analysed and presented in the main publication before unblinding of study data. Statistical analysis plans limit selective reporting bias. This statistical analysis plan will increase the validity of the final trial results.

**Trial registration:**

ClinicalTrials.gov NCT03595098. July 23, 2018

**Supplementary Information:**

The online version contains supplementary material available at 10.1186/s13063-022-06799-4.

## Introduction

Obsessive-compulsive disorder (OCD) is a psychiatric disorder which affects up to 3% of children and adolescents [1]. OCD is associated with reduced quality of life [2] and significant impairment at home, in school, and with friends [3, 4]. OCD is generally treated with behavioural therapy or cognitive behavioural therapy (CBT), which, in more severely affected patients, can be combined with antidepressant medication [5, 6].

CBT for OCD may have the same effects in children and adolescents as in adults [7, 8], two-thirds of whom respond at end of treatment [7]. Psychotherapy, including CBT, is often considered harmless by patients and therapists in comparison to psychopharmacological treatment. This assumption may stem from the limited scientific reports on psychotherapy-related adverse events and reactions [9]. Nevertheless, lasting harms to psychotherapy have been reported in 5% of adult patients [10].

We recently conducted a systematic review which assessed the effectiveness of CBT compared with pharmacotherapy with selective serotonin reuptake inhibitors or with no intervention [11]. We designed the TECTO trial [12] as a randomised clinical trial for children and adolescents aged 8 to 17 years, which aims to compare the benefits and harms of family-based CBT (FCBT) versus family-based psychoeducation with relaxation training (FPRT) as an active control intervention. This statistical analysis plan outlines the planned statistical analyses for the TECTO trial, depicts a pre-programmed statistical report, and discusses the comparison of the experimental and control interventions.

## Methods

The TECTO trial is an investigator-initiated, independently funded, single-centre, parallel-group, superiority randomised clinical trial [12]. The trial methodology and design have been described in detail elsewhere [12]. Briefly, patients are recruited in a single centre in the Capital Region of Denmark. Here, we offer both intervention groups 14 therapy sessions of 75 min each during a period of 16 weeks. The experimental intervention consists of sessions of FCBT, whereas the sessions in the control group consist of FPRT. The randomised TECTO trial is combined with several longitudinal case-control sub-studies that will be described elsewhere. The trial was registered on ClinicalTrials.gov (identification no. NCT03595098; 23 July 2018) before the inclusion of the first participant. Patients are eligible if they comply with the inclusion and exclusion criteria below.

The following are the inclusion criteria:

- OCD diagnosis as the primary diagnosis, meeting the criteria for ICD-10 F42, verified with a semi-structured clinical interview (Kiddie-Schedule for Affective Disorders and Schizophrenia – Present and Lifetime Version, K-SADS-PL) [13]
- Children’s Yale–Brown Obsessive-Compulsive Scale (CY-BOCS) [14] entry score ≥ 16, a cutoff score used in previous studies [15, 16]
- 8 to 17 years of age (both inclusive)
- Signed informed consent from legal guardians

The following are the exclusion criteria:

- Comorbid illness that contraindicates trial participation, e.g. pervasive developmental disorder not including Asperger’s syndrome, schizophrenia/paranoid psychosis, mania or bipolar disorder, depressive psychotic disorders, or substance dependence syndrome
- Intelligence quotient < 70
- Treatment with CBT, psychoeducation with relaxation training, serotonin reuptake inhibitors, or other antidepressant or antipsychotic medication within the last 6 months prior to trial entry.

### Randomisation and blinding

Participants are randomised at the allocation ratio of 1:1 using a web-based randomisation system handled centrally by the Copenhagen Trial Unit using a concealed computer-generated allocation sequence with a varying block size concealed from investigators. Randomisation is stratified by age (8 to 12 compared to 13 to 17 years) and CY-BOCS total score at baseline (16 to 23 compared to ≥ 24 points). Due to the nature of the intervention, blinding of participants and clinicians is not possible. However, trained investigators are blinded to the allocation during outcome assessments, and the participants and their legal guardians/caregivers are instructed not to disclose allocation. For further details on blinding see the “Statistical reports” section.

### Trial interventions

The key components of the manualised FCBT are exposure and response prevention (ERP), family involvement, psychoeducation, and homework assignments. ERP refers to gradual exposure to distress/anxiety-provoking situations that trigger obsessional thinking and subsequent abstinence from compulsive behaviour. In the TECTO trial, ERP is combined with cognitive techniques, such as normalising intrusive thoughts. The manualised FCBT is described in detail in the design article [12].

FPRT is the active control intervention. We adapted the FPRT manual to have an equal number of sessions of similar duration as the experimental intervention [15]. FPRT consists of relaxation training, with activation and relaxation of individual muscles and muscle groups and breathing exercises, family involvement, psychoeducation, and homework assignments. The manualised FPRT is described in detail in the design article [12].

### Outcomes

The primary outcome is the severity of OCD symptoms assessed using the structured interview CY-BOCS at week 16. The two secondary outcomes are health-related quality of life (HRQoL), assessed using the questionnaire KIDSCREEN-52 [17], and adverse events, assessed using the Negative Effects Questionnaire (NEQ) [18]. The primary and one secondary outcomes are assessed at baseline (not NEQ), week 4, week 8, and at the end of intervention (week 16). The KIDSCREEN-52 has multiple subscores and will be reported as global HRQoL based on the shorter questionnaire KIDSCREEN-10, which can be derived from the 52-question version [19]. The two secondary outcomes have more than 80% power (*see below*). The several exploratory outcomes are considered hypothesis-generating and will be interpreted with caution. All outcomes are presented in Table 1.

**Table 1:** Outcomes in the TECTO trial

Outcomes	Type of data
**Primary outcome**	
OCD symptoms *Children’s Yale–Brown Obsessive Compulsive Scale (CY-BOCS)* [14]	Continuous *Assessed at weeks 0, 4, 8, and 16*
**Secondary outcomes**	
Health-related quality of life^a^ *KIDSCREEN-10* [17]	Continuous *Assessed at weeks 0, 4, 8, and 16*
Adverse events *Negative Effects Questionnaire (NEQ) with 32 items* [18]	Continuous *Assessed at weeks 4, 8, and 16*
**Exploratory outcomes**	
Serious adverse events	Dichotomous^c^ *Assessed for weeks 0 to 16*
Assessment of childhood OCD^b^ *Child Obsessive-Compulsive Impact Scale-Revised (COIS-R)* [20]	Continuous *Assessed at weeks 0, 4, 8, and 16*
Severity of psychopathology *Clinical Global Impressions Severity (CGI-S)* [21]	Continuous *Assessed at weeks 0, 4, 8, and 16*
Change of psychopathology after initiation of treatment *Clinical Global Impressions Improvement (CGI-I)* [21]	Continuous *Assessed at weeks 4, 8, and 16*
Severity of psychiatric disturbance and social disability *Children’s Global Assessment Scale (CGAS)* [22]	Continuous *Assessed at weeks 0 and 16*
Response rate *Defined as a CY-BOCS score reduction of at least 30% from baseline*	Dichotomous *Assessed at weeks 0 and 16*
Remission rate *Defined as no longer meeting the diagnostic criteria for OCD, ICD-10 F42 assessed with K-SADS-PL* [13]	Dichotomous *Assessed at week 16*
Suicidality *Suicidality items sum-score assessed with K-SADS-PL* [13]	Continuous *Assessed at week 16*
Obsessive-compulsive traits^b^ *Toronto Obsessive-Compulsive Scale (TOCS)* [23]	Continuous *Assessed at weeks 0 and 16*
Parental accommodation to children’s obsessions and compulsions *Family Accommodation Scale – Parent Report (FAS-PR)* [24]	Continuous *Assessed at weeks 0, 4, 8, and 16*
Parental stress *Parental Stress Scale (PSS)*	Continuous *Assessed at weeks 0, 4, 8, and 16*

*OCD* obsessive-compulsive disorder, *K-SADS-PL* Kiddie-Schedule for Affective Disorders and Schizophrenia - Present and Lifetime Version

^a^Results from participants will be analysed as a secondary outcome, while results from parents and/or legal guardians will be analysed as an exploratory outcome

^b^Results from participants and parents and/or legal guardians will be presented separately

^c^If there are multiple serious adverse events per participant, they will also be assessed as a count outcome

The KIDSCREEN-10, the Child Obsessive Compulsive Disorder Impact Scale revised (COIS-R), and the Toronto Obsessive-Compulsive Rating Scale (TOCS) are questionnaires which are completed by participants and the parents and/or legal guardians, while the questionnaires Family Accommodation Scale (FAS) and Parental Stress Scale (PSS) will only be answered by caregivers. For questionnaires answered by participants and caregivers, the answers from the caregivers will be analysed as exploratory outcomes. In participants for whom two caregivers complete the questionnaires, we use the one with the most complete dataset. If both are equally complete the mean value of the two will be used for analyses. Parental participation is described in more detail in the design paper [12].

The CY-BOCS, the KIDSCREEN, the COIS-R, the Clinical Global Impression, Severity/Improvement (CGI-S/I), the NEQ, and the FAS will also be assessed at week 40 and reported in a separate manuscript. The analytic principles will follow those described in this statistical analysis plan.

### Sample size calculation

This randomised clinical trial is designed to evaluate superiority when comparing the two intervention groups. The minimum relevant difference (MIREDIF) of the primary outcome, CY-BOCS, is 4 points, and we expect a standard deviation (SD) of 8 points [11]. Using the two-sample *t*-test sample size calculation with 80% power and an alpha of 0.05, we aim to include at least 64 participants in each group for a total of at least 128 participants.

### Power calculation

The secondary outcome KIDSCREEN-10 is presented using the general HRQoL index [19, 25]. For KIDSCREEN-10, we pragmatically chose a MIREDIF of 1.0 point corresponding to 10% of the maximum scale and expected an SD of 1.5 points [19], which results in 96% power [26]. The secondary outcome NEQ is presented as the frequency of negative effects, a summed score of the 20 questions from the 20 items questionnaire of NEQ [25, 27]. For the secondary outcome NEQ, we pragmatically chose a MIREDIF of 2.0 points corresponding to 10% of the maximum scale and expected an SD of 3.5 points [27], which results in 89% power [26].

### General analysis principles

Statistical analyses will be performed using the latest stable version of R (R Core Team, Vienna, Austria), SAS (SAS Institute, NC, USA), and/or Stata (StataCorp LLC, TX, USA). We will use at least two different softwares for each analysis. All valid measurements from all randomised participants will be included in all analyses (the intention-to-treat principle). Dropouts and discontinuation of treatment will be reported. The baseline characteristics will be presented for each group (Table 2).

**Table 2:** Baseline characteristics based on simulated data

		A (*n* = 64)	B (*n* = 64)
Age (years)	Mean (SD)	16 (0.6)	15.9 (0.6)
Gender	Female	16 (25.0)	20 (31.2)
	Male	18 (28.1)	20 (31.2)
	Transgender	13 (20.3)	12 (18.8)
	Others	17 (26.6)	12 (18.8)
Nationality	Danish	28 (43.8)	32 (50.0)
	Others	36 (56.2)	32 (50.0)
Parental education level (ISCED)	Mean (SD)	3.8 (2.6)	3.8 (2.6)
Parental nationality	Danish	25 (39.1)	18 (28.1)
	Danish and others	19 (29.7)	27 (42.2)
	Others	20 (31.2)	19 (29.7)
Full-scale IQ	Mean (SD)	94.2 (15.6)	95.5 (14.7)
OCD subtype	Mixed obsessional thoughts and acts	21 (32.8)	21 (32.8)
	Predominantly compulsive acts	25 (39.1)	24 (37.5)
	Predominantly obsessional thoughts or ruminations	18 (28.1)	19 (29.7)
Comorbidities^a^	Depressive disorders	32 (50.0)	36 (56.2)
	Anxiety disorders	33 (51.6)	23 (35.9)
	Adjustment disorders	26 (40.6)	37 (57.8)
	Eating disorders	36 (56.2)	30 (46.9)
	Personality disorders	38 (59.4)	30 (46.9)
	Asperger’s syndrome	41 (64.1)	35 (54.7)
	Hyperkinetic disorders	34 (53.1)	29 (45.3)
	Conduct disorders	27 (42.2)	24 (37.5)
	Tics/Tourette’s syndrome	39 (60.9)	32 (50.0)
	Elimination disorders	33 (51.6)	41 (64.1)
Baseline psychopathology			
CY-BOCS	Mean (SD)	20.1 (11)	20.3 (11.4)
KIDSCREEN	Mean (SD)	− 1.9 (1.5)	− 2.1 (1.4)
COIS-R	Mean (SD)	14.5 (10.4)	14.8 (9.8)
CGI-S	Mean (SD)	15 (10.4)	15.9 (10)
CGAS	Mean (SD)	22.7 (13.4)	16.6 (11.9)
TOCS	Mean (SD)	20.1 (13.8)	21 (11.9)
SRS	Mean (SD)	23.1 (23.1)	21 (1.3)
Family characteristics			
FES—relationship dimensions	Cohesion	54.5 (26.6)	51.1 (33.1)
	Expressiveness	51.2 (31.2)	56.3 (30.2)
	Conflict	50.9 (27.1)	51.3 (29.8)
FES—personal growth dimensions	Independence	53.1 (28.7)	49.5 (28.8)
	Achievement orientation	44.3 (31.6)	50.4 (29.3)
	Intellectual-cultural orientation	51.6 (28.2)	50.5 (29.2)
	Active-recreational orientation	48.8 (29.3)	50.5 (26.8)
	Moral-religious emphasis	50.2 (28.8)	51.4 (30.9)
FES—system maintenance dimensions	Organisation	46.1 (28.2)	47.2 (29.1)
	Control	46.6 (28.8)	53.3 (27.8)
FAS	Mean (SD)	20.2 (13.7)	23.4 (13.3)
PSS	Mean (SD)	22.3 (12.6)	20.5 (13.1)

The education level of the parent with the highest education is used (using ISCED). For PSS and FES, the average of the parents who responded is presented

*CY-BOCS* Children’s Yale–Brown Obsessive Compulsive Scale, *COIS-R* Child Obsessive-Compulsive Impact Scale-Revised, *CGI-S* Clinical Global Impressions Severity, *CGAS* Children’s Global Assessment Scale, *TOCS* Toronto Obsessive-Compulsive Scale, *FES* Family Environment Scale, *PSS* Parental Stress Scale, *FAS* Family Accommodation Scale for Obsessive-Compulsive Disorder, *SD* standard deviation, *SRS* Social Responsiveness Scale

^a^Comorbidities are just examples, and actual comorbidities from the trial will be added here

### Statistical analysis

#### Continuous outcomes

**Fig. 1:**
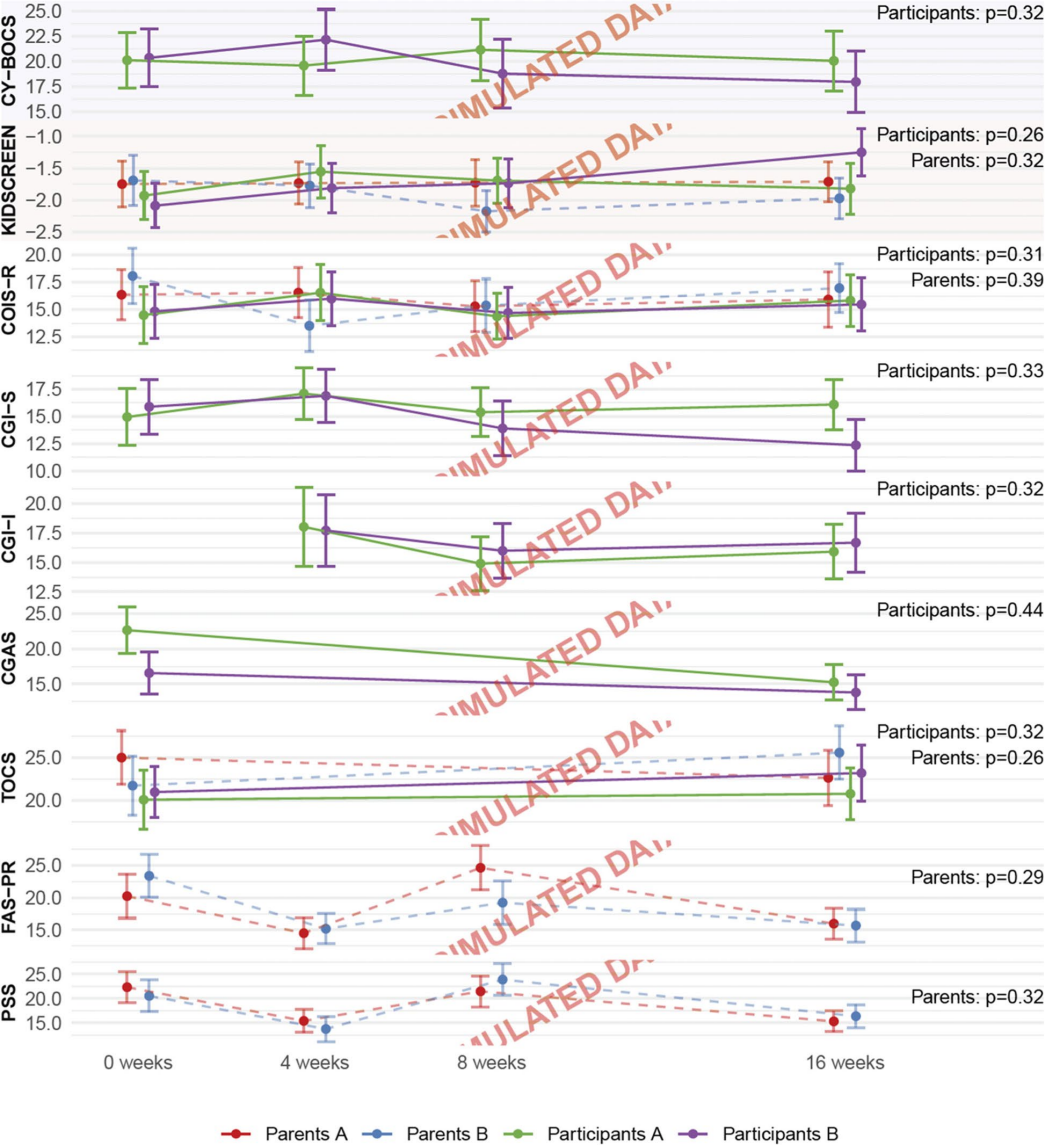
Psychopathology and family burden based on simulated data. Presentation of the timeline of the primary outcome (light blue background), secondary outcomes (light red background), and exploratory outcomes, with results from the analyses, with baseline correction for the outcomes assessed at baseline, with *p*-values for the secondary outcomes corrected for multiplicity. CY-BOCS, Children’s Yale–Brown Obsessive Compulsive Scale; COIS-R, Child Obsessive-Compulsive Impact Scale-Revised; CGI-S, Clinical Global Impressions Severity; CGI-I, Clinical Global Impressions Improvement; CGAS, Children’s Global Assessment Scale; TOCS, Toronto Obsessive-Compulsive Scale; FAS, Family Accommodation Scale for Obsessive-Compulsive Disorder; PSS, Parental Stress Scale

Continuous outcomes will be presented as means and 95% confidence intervals in figures and as means and SD in a supplemental table (Fig. 1, Supplemental Material). Continuous outcomes will be analysed using linear regression adjusted for the stratification variables and baseline value of the continuous outcome, if any.

#### Count data outcomes

Count data outcomes will be presented as medians and interquartile ranges in figures and in a supplemental table. Count data will be analysed using the Wilcoxon rank sum exact test and median differences and corresponding confidence intervals will be presented using Hodges-Lehman [28].

#### Dichotomous outcomes

Dichotomous outcomes will be presented as proportions for each group and will be analysed using logistic regression with stratification variables as fixed effects. We will estimate the marginal effects to obtain relative risks (RRs) and 95% confidence intervals of the RRs (based on “nlcom” from Stata (StataCorp, TX, USA)) and/or g-computations in R (R Core Team, Vienna, Austria) [29]. Numbers needed to treat and numbers needed to harm will be presented when relevant.

#### COVID-19 pandemic as a moderator

Large parts of the Danish society were closed on 16 March 2020 and onwards during the trial period in varying degrees [30]. These shutdowns are believed to have negatively affected the psychiatric population including children and adolescents with OCD [31, 32]. Therefore, we will carry out additional exploratory sensitivity analyses by comparing the effects of the interventions before and after the lockdown period (interaction between the treatment variable and inclusion before or after the lockdown date, March 16, 2020). The significance level for the interaction is 0.05. Participants who were included before the lockdown and had follow-ups after will be excluded from this analysis.

#### Therapy factors as moderators

The confidence in treatment, motivation for treatment, the therapeutic alliance, and compliance might influence the efficiency of the interventions. Consequently, we will carry out additional exploratory analyses by comparing the effects of the interventions based on (1) the confidence in treatment (assessed by the therapist after the first session; 7-point Likert scale), (2) motivation for treatment (average of assessments from week 0, 1, 4, 8, and 14; 7-point Likert scale), (3) the therapeutic alliance (average of assessments from week 1, 4, 8, and 16; Therapeutic Alliance Scale for Children–revised; TASC-R [30, 31]), and (4) compliance (assessed as the quality of the participant’s homework after every session throughout treatment period; 7-point Likert scale). Effect modification is assessed as the interaction between the treatment variable and these four therapy factors. The analyses will be carried out as complete case analyses.

### Correction for multiplicity

We assess only one primary outcome and consider all other outcome results as hypothesis-generating only. Therefore, we have used a two-sided alpha of 5% as the acceptable risk of type I error in the sample size and power estimations.

### Handling of missing data

Missing data for the primary and secondary outcomes will be handled per the recommendations by Jakobsen and colleagues [33]. In short, we will consider the use of multiple imputation and/or present best-worst and worst-best case scenarios. If multiple imputation is deemed necessary, we will include the assessment of CY-BOCS assessed at week 8 as a covariate in the imputation of the primary outcome and KIDSCREEN-10 and NEQ assessed at week 8 for the secondary outcomes. Furthermore, we will include baseline assessments when available. The exploratory outcomes will be analysed using complete participant analysis.

#### Exploratory mixed-effect model analysis

Using unimputed data from the primary outcome, CY-BOCS, we will carry out an exploratory mixed-effects model for repeated measures with intervention group and time as fixed effects and participants as random effects. The model will include all the measurements of CY-BOCS from week 0 until follow-up. This analysis investigates if there are group-specific differences over time.

### Assessments of underlying statistical assumptions

We will systematically assess the statistical assumptions underlying each statical method per the recommendations by Nørskov and colleagues [34, 35]. In short, for all regression analyses, we will test for major interactions between each covariate and the intervention variable. When assessing for major interactions, we will, in turn, include each possible first-order interaction between included covariates and the intervention variable. For each combination, a significant interaction is only evident if the interaction is statistically significant after Bonferroni adjusted thresholds (0.05 divided by the number of possible interactions) and if the interaction shows a clinically important effect. If an interaction is evident, we will consider presenting an analysis separately for each and an overall analysis including the interaction term in the model [34, 35].

The variables included in the linear regression models will be visually assessed for normal distribution using histograms and quantile-quantile plots of the residuals, and for homogeneity using residuals plotted against covariates and fitted values, with the possibility of a logarithmic transformation or applying robust standard errors to minimise deviations from the model [35].

To assess relevant overdispersion, we divide the deviance by the degrees of freedom for the logistic regression model. For dichotomous outcomes with few or zero events identified (substantially lower than the rule of thumb of 10 events), the analyses will be carried out using Fisher’s exact test**.** The robustness of the confidence intervals and *p*-values might be affected by the small sample size and these will be interpreted with caution [35].

#### Statistical reports

To expedite and bolster analyses once the data of the TECTO trial are collected and cleaned, we have developed a statistical report based on simulated data. The simulated statistical report is available on Zenodo (https://zenodo.org/record/6340142#.Yih__3qZOUk; European Organization for Nuclear Research, Genevé, Switzerland) and is submitted as Supplemental material. The statistical report with the analyses chosen for the manuscript is being tracked using a version control system (https://github.com/lilleoel/CTU_TECTO, GitHub, San Francisco, CA, USA). After completion of the trial, two independent statisticians will analyse the blinded data, where “A” and “B” refer to the two groups. The statisticians will independently test for assumptions and choose the correct analysis for each outcome. The chosen analyses are based on this statistical analysis plan and the pre-programmed statistical report. The results will be presented in two independent reports, which will be compared by the coordinating investigator, the two statisticians, and the steering committee. Based on consensus from this group, the final statistical report will be used to write two abstracts by the steering committee: One assuming “A” is the experimental group and “B” is the control group—and one assuming the opposite. These abstracts will use the results from the blinded final report, and when the blinding is broken, the “correct” abstract will be chosen and the conclusions in this abstract will not be revised. Furthermore, all three statistical reports will be published as supplementary material.

## Results

**Fig. 2:**
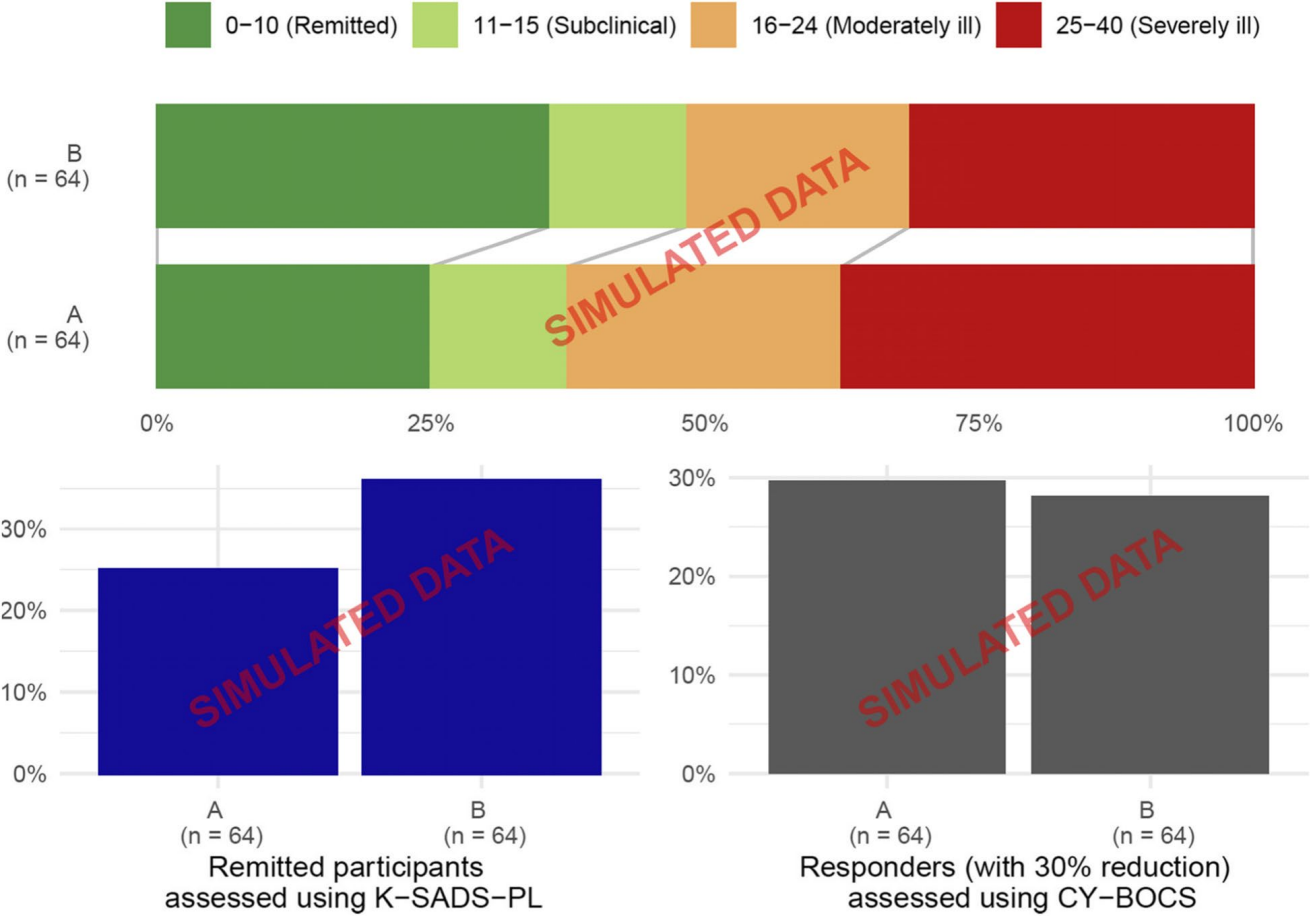
Response status at 16 weeks based on simulated data. The response status at follow-up, by categorising CY-BOCS into severity and the proportion of remitted participants assessed using K-SADS-PL, and responders defined as a 30% reduction in CY-BOCS score compared to baseline. CY-BOCS, Children’s Yale–Brown Obsessive Compulsive Scale; K-SADS-PL, Kiddie-Schedule for Affective Disorders and Schizophrenia - Present and Lifetime Version

**Fig. 3:**
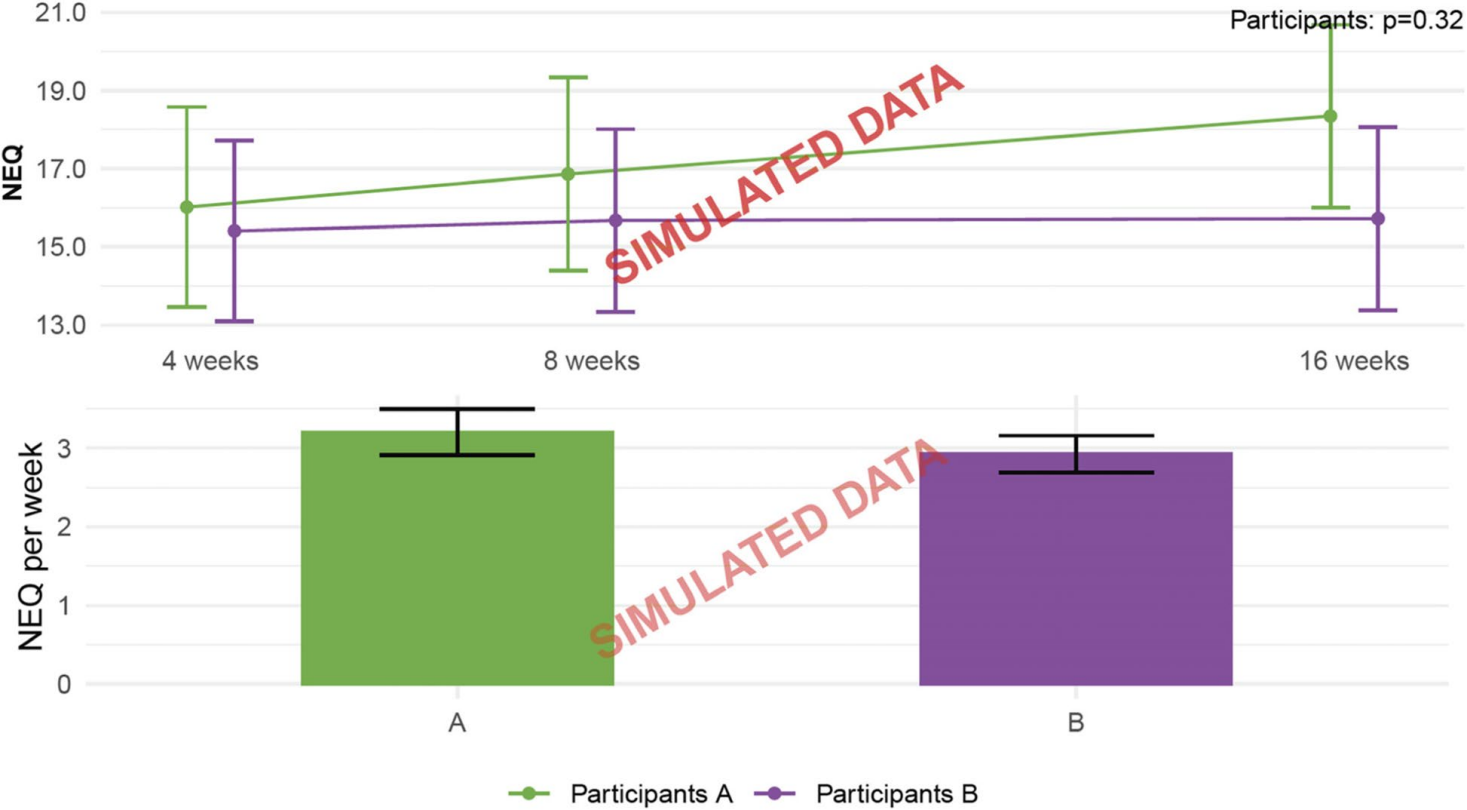
The Negative Effects Questionnaire based on simulated data. Adverse events assessed using the Negative Effects Questionnaire (NEQ). Assessment at weeks 4 and 8 reflect the last 4 weeks, while the assessment at week 16 reflects the last 8 weeks. NEQ per week is calculated using the average weekly score

See Table 2 and Figs. 1, 2, and 3 with simulated data prepared for the final manuscript.

## Discussion

This paper presents a predefined description of the statistical analyses of the TECTO trial, which will limit bias and data-driven interpretations and conclusions. The detailed plan of handling missing data will further increase the validity of our results.

The purpose of the chosen control intervention in this trial is to mimic the experimental intervention but precludes what is the most likely the active ingredient of the experimental intervention [12]. Choosing a control intervention for psychiatric trials introduces a potential bias, whereas waitlist and treatment-as-usual have the highest probability of achieving a statistically significant positive experimental effect [36]. Our chosen protocolised active control intervention may limit our ability to achieve statistical significance, but the design will inform us as to whether the selected active ingredient (ERP) in FCBT is effective.

### Strengths

The primary strengths are the predefined statistical analysis plan, and publication of a version-controlled, pre-programmed statistical report before any data is available. Since different statistical software sometimes produce different results, we plan on using at least two different statistical software programs for each analysis. We will thoroughly report if such differences occur and identify the most correct result.

This statistical analysis plan will minimise the selective reporting bias [37–39]. The plan secures methodological transparency and enables reproducibility of our results. The details of this statistical analysis plan should be reported in sufficient detail so independent statisticians may reproduce the statistical analyses [40]. Publicly available statistical analysis plans registered before the results are known are necessary [41], and together with the details acquired by using simulated data, we can reduce the risk of selective reporting bias.

The strict outcome hierarchy with one primary outcome, two secondary outcomes with a power of 96% and 89%, and several exploratory outcomes is another strength. We have calculated the sample size for the primary outcome, and we will base our primary conclusions from the trial on this primary outcome. We have selected only two secondary outcomes, for which we have defined MIREDIF and calculated power according to a risk of type I error. Conversely, the exploratory outcomes will be purely hypothesis-generating. This outcome hierarchy reduces the risk of type 1 errors in the interpretation of trial results [42].

### Limitations

The primary limitation is the high risk of missing data in psychiatric clinical trials [43, 44]. We have tried to address this by explicitly stating our plan for the multiple imputation, which we may be compelled to carry out. Another limitation is the superiority design, in which firm conclusions can only be drawn if differences between the groups are identified for the primary outcome. However, non-inferiority and equivalence trials are limited with respect to addressing similarities and do not address whether one treatment is better than another. Even without yielding statistical significance, the results of our superiority trial still contribute to the accumulating evidence across trials and can be included in systematic reviews and meta-analyses.

Furthermore, the TECTO trial includes many exploratory outcomes which increase the risk of type I errors. However, we will interpret these with caution, and the results will only be hypothesis-generating.

## Conclusion

This statistical analysis plan for the TECTO trial includes a detailed predefined description of how data will be analysed and presented in the main publication. We have included descriptions of the statistical considerations and attached a pre-programmed, version-controlled statistical report with simulated data. Statistical analysis plans limit selective reporting bias. This statistical analysis plan will increase the validity of the final trial results.

## Supplementary Information

**Additional file 1: Table 1.** Participant characteristics. **Figure 1.** CONSORT flow diagram. **Figure 2.** Psychopathology and family burden. **Figure 3.** Response status at 16 weeks. **Figure 4.** Negative effects questionnaire. **Supplemental Table 1.** Detailed comorbidities. **Supplemental Table 2.** Psychopathology and family burden. **Supplemental Table 3.** Kidscreen-52. Supplemental Timeline. Supplemental Assumptions.

## Data Availability

The datasets generated and/or analysed during the current trial are available at https://zenodo.org/record/XXX and https://github.com/lilleoel/CTU_TECTO

## Abbreviations

CBT: Cognitive behavioural therapy
CGAS: Children’s Global Assessment Scale
CGI-I: Clinical Global Impressions Improvement
CGI-S: Clinical Global Impressions Severity
COIS-R: Child Obsessive-Compulsive Impact Scale-Revised
CY-BOCS: Children’s Yale–Brown Obsessive-Compulsive Scale
ERP: Exposure and response prevention
FAS: Family Accommodation Scale for Obsessive-Compulsive Disorder
FCBT: Family-based cognitive behavioural therapy
FES: Family Environment Scale
FPRT: Family-based psychoeducation/relaxation training
HRQoL: Health-related quality of life
K-SADS-PL: Kiddie-Schedule for Affective Disorders and Schizophrenia – Present and Lifetime Version
MIREDIF: Minimum relevant difference
NEQ: Negative Effects Questionnaire
OCD: Obsessive-compulsive disorder
PSS: Parental Stress Scale
SD: Standard deviation
TOCS: Toronto Obsessive-Compulsive Scale
