# Supplementary material for: Family-based cognitive behavioural therapy versus family-based relaxation therapy for obsessive-compulsive disorder in children and adolescents (the TECTO trial): a statistical analysis plan for the randomised clinical trial
Source: Trials. 2022 Oct 6;23:854. doi: 10.1186/s13063-022-06799-4 (PMC9535232; doi:10.1186/s13063-022-06799-4)

Statistical report for TECTO trial

Using simulated data

12 juni 2022

Contents

Table 1 - Participant characteristics **2**

Figure 1 - CONSORT flow diagram **4**

Figure 2 - Psychopathology and family burden **5**

Figure 3 - Response status at 16 weeks **6**

Figure 4 - Negative effects questionnaire **7**

Supplemental Table 1 - Detailed comorbidities **8**

Supplemental Table 2 - Psychopathology and family burden **9**

Supplemental Table 3 - Kidscreen-52 **10**

Supplemental Table 4 - SAE/SAR/SUSAR **11**

Supplemental Timeline **12**

Supplemental Assumptions **13**

- CY-BOCS (primary) 13
- KIDSCREEN-10 (secondary) 14
- NEQ (secondary) 14
- COIS-R (exploratory) 15
- CGI-S (exploratory) 15
- CGI-I (exploratory) 16
- CGAS (exploratory) 16
- TOCS (exploratory) 17

Table 1 - Participant characteristics

		A (n=64)	B (n=64)
Age (years)	mean (sd)	16 (0.6)	15.9 (0.6)
Gender	Female	16 (25.0)	20 (31.2)
	Male	18 (28.1)	20 (31.2)
	Other	13 (20.3)	12 (18.8)
	Transgender	17 (26.6)	12 (18.8)
Nationality	Other	28 (43.8)	32 (50.0)
	Danish	36 (56.2)	32 (50.0)
Parental education level (ISCED)	mean (sd)	3.8 (2.6)	3.8 (2.6)
Parental nationality	Other	25 (39.1)	18 (28.1)
	Danish and other	19 (29.7)	27 (42.2)
	Danish	20 (31.2)	19 (29.7)
Full-scale IQ	mean (sd)	94.2 (15.6)	95.5 (14.7)
OCD-subtype	Mixed obsessional thoughts and acts	21 (32.8)	21 (32.8)
	Predominantly compulsive acts	25 (39.1)	24 (37.5)
	Predominantly obsessional thoughts or ruminations	18 (28.1)	19 (29.7)
Comorbidities	Depressive disorders	32 (50.0)	36 (56.2)
	Anxiety disorders	33 (51.6)	23 (35.9)
	Adjustment disorders	26 (40.6)	37 (57.8)
	Eating disorders	36 (56.2)	30 (46.9)
	Personality disorders	38 (59.4)	30 (46.9)
	Aspergers Syndrome	41 (64.1)	35 (54.7)
	Hyperkinetic disorders	34 (53.1)	29 (45.3)
	Conduct disorders	27 (42.2)	24 (37.5)
	Tics/Tourettes syndrome	39 (60.9)	32 (50.0)
	Elimination disorders	33 (51.6)	41 (64.1)

Baseline psychopathology		A (n=64)	B (n=64)
CY-BOCS	mean (sd)	20.1 (11)	20.3 (11.4)
KIDSCREEN	mean (sd)	-1.9 (1.5)	-2.1 (1.4)
COIS-R	mean (sd)	14.5 (10.4)	14.8 (9.8)

Baseline psychopathology		A (n=64)	B (n=64)
CGI-S	mean (sd)	15 (10.4)	15.9 (10)
CGAS	mean (sd)	22.7 (13.4)	16.6 (11.9)
TOCS	mean (sd)	20.1 (13.8)	21 (11.9)

Family characteristics		A (n=64)	B (n=64)
FES - Relationship Dimensions	Cohesion	54.5 (26.6)	51.1 (33.1)
	Expressiveness	51.2 (31.2)	56.3 (30.2)
	Conflict	50.9 (27.1)	51.3 (29.8)
FES - Personal Growth Dimensions	Independence	53.1 (28.7)	49.5 (28.8)
	Achievement Orientation	44.3 (31.6)	50.4 (29.3)
	Intellectual-Cultural Orientation	51.6 (28.2)	50.5 (29.2)
	Active-Recreational Orientation	48.8 (29.3)	50.5 (26.8)
	Moral-Religious Emphasis	50.2 (28.8)	51.4 (30.9)
FES - System Maintenance Dimensions	Oranization	46.1 (28.2)	47.2 (29.1)
	Control	46.6 (28.8)	53.3 (27.8)
FAS-PR	mean (sd)	20.2 (13.7)	23.4 (13.3)
PSS	mean (sd)	22.3 (12.6)	20.5 (13.1)

The education-level from the parent with the highest education is used (using ISCED). For PSS, FES, and SRS the average of the parents who responded is presented.

CY-BOCS: Children's Yale–Brown Obsessive Compulsive Scale; COIS-R: Child Obsessive-Compulsive Impact Scale-Revised; CGI-S: Clinical Global Impressions Severity; CGAS: Children's Global Assessment Scale; TOCS: Toronto Obsessive-Compulsive Scale; FES: Family Environment Scale; PSS: Parental stress scale; FAS-PR: Family Accommodation Scale for Obsessive-Compulsive Disorder.

Figure 1 - CONSORT flow diagram

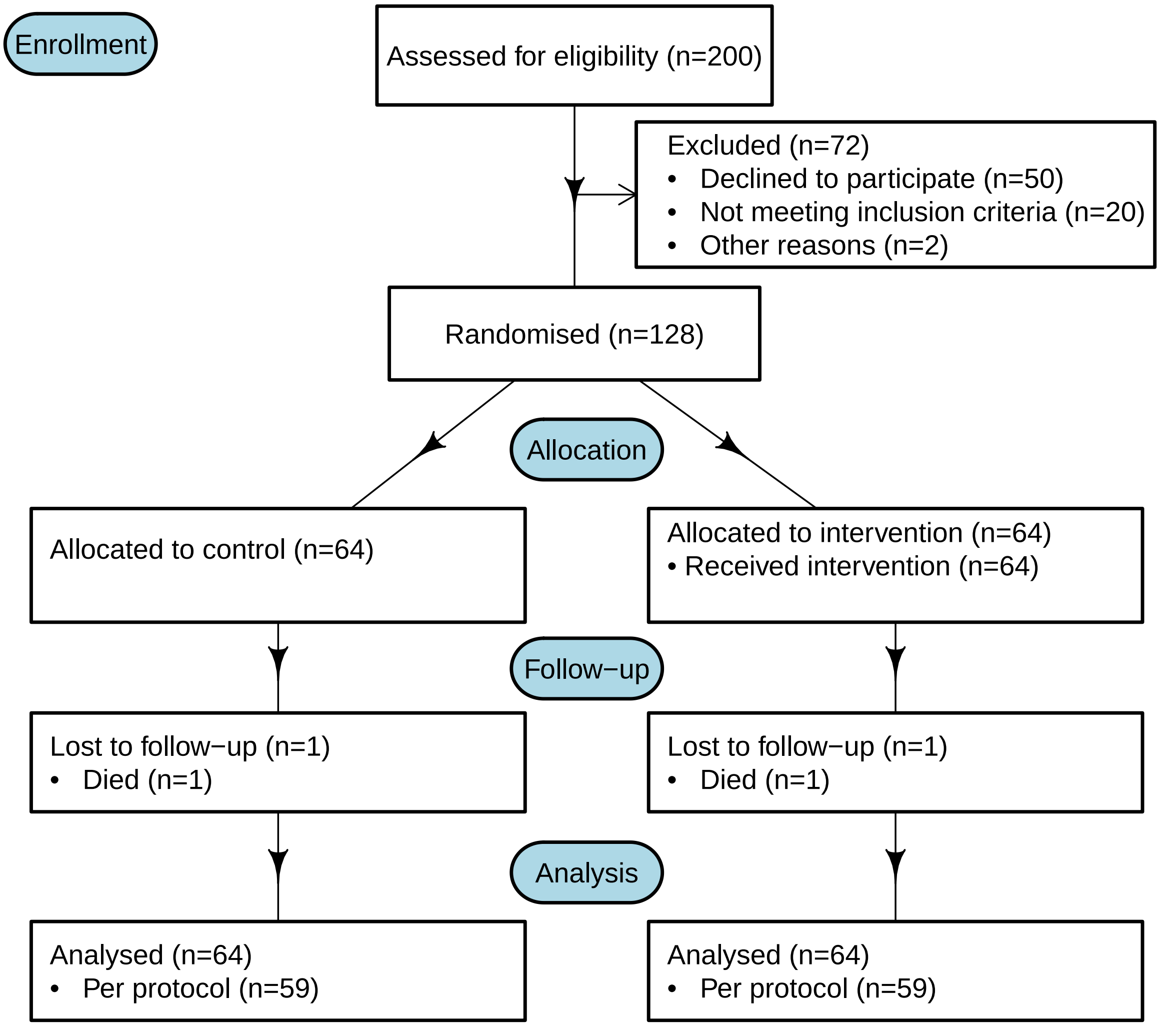

Figure 2 - Psychopathology and family burden

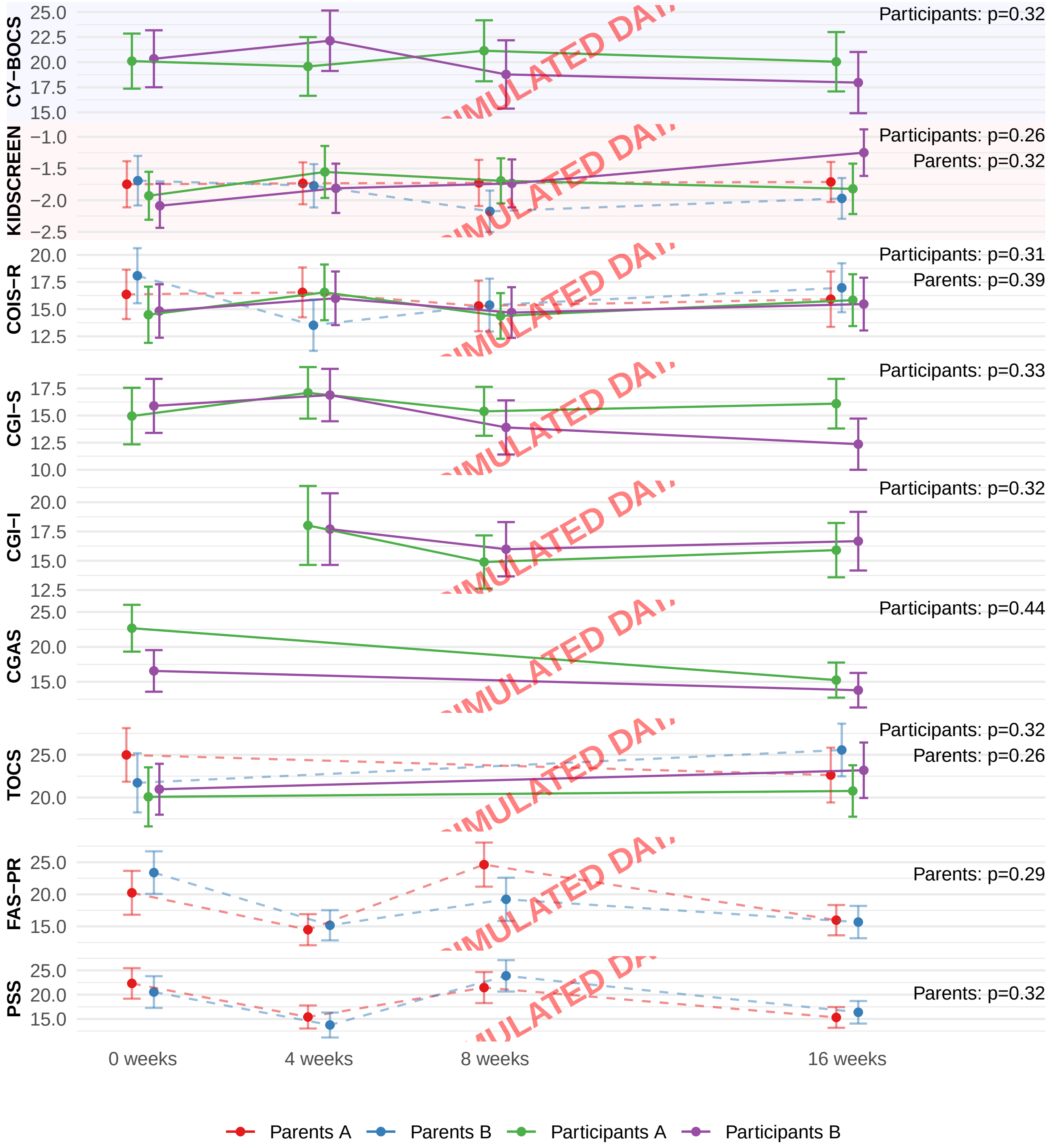

CY-BOCS: Children's Yale–Brown Obsessive Compulsive Scale; COIS-R: Child Obsessive-Compulsive Impact Scale-Revised; CGI-S: Clinical Global Impressions Severity; CGI-I: Clinical Global Impressions Improvement; Children's Global Assessment Scale; TOCS: Toronto Obsessive-Compulsive Scale; FAS-PR: Family Accommodation Scale for Obsessive-Compulsive Disorder; PSS: Parental stress scale.

Figure 3 - Response status at 16 weeks

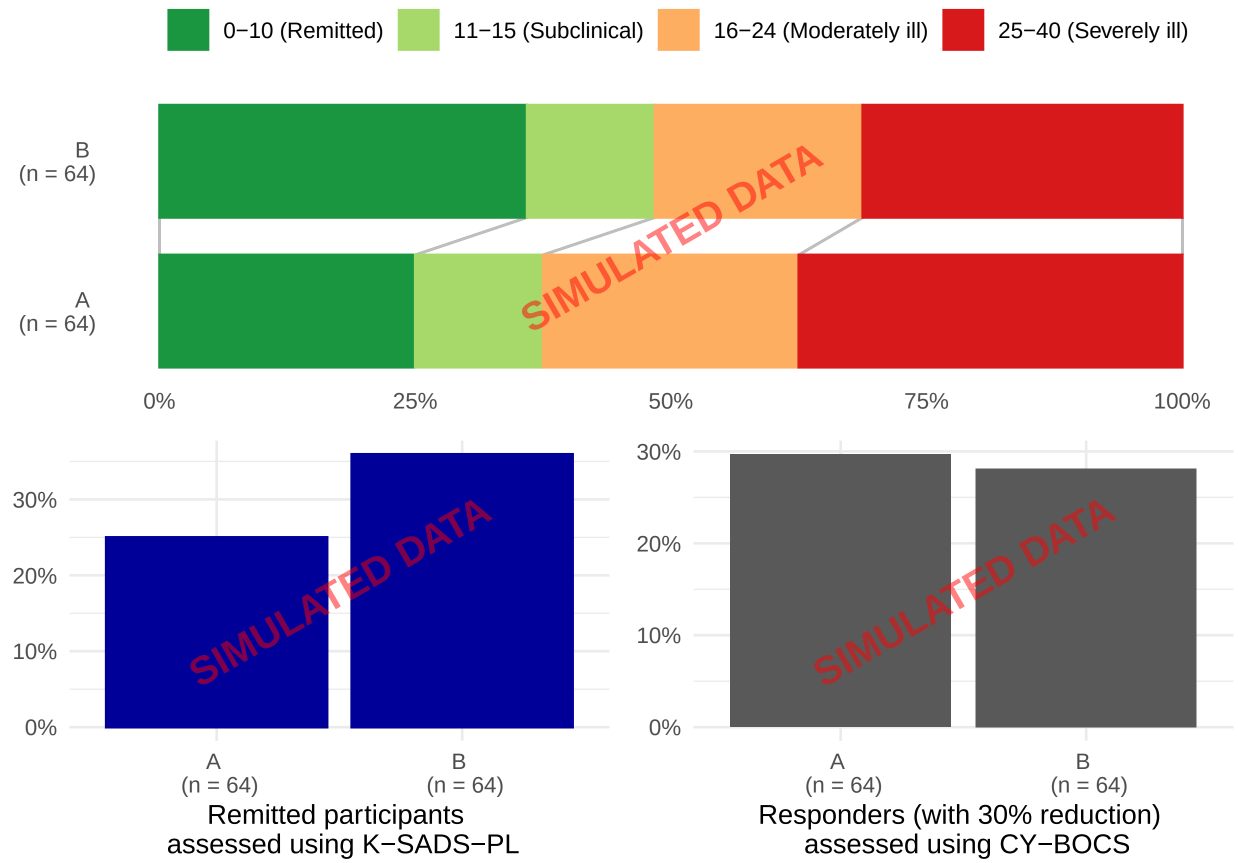

Figure 4 - Negative effects questionnaire

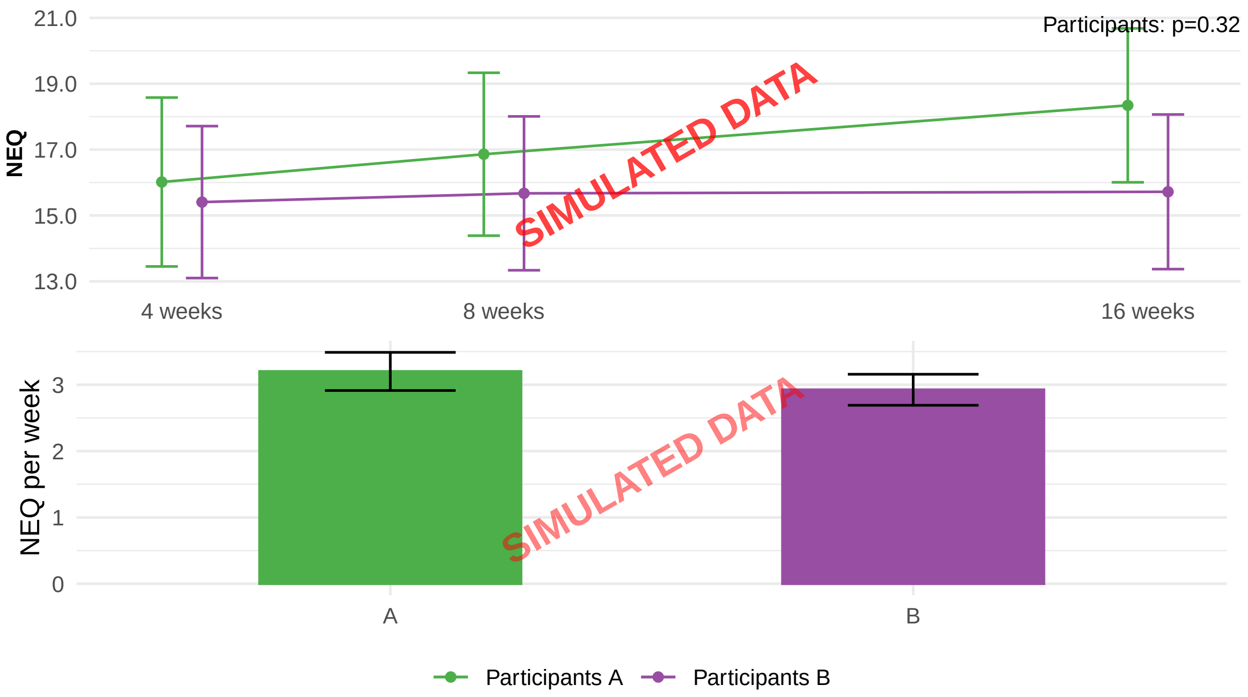

Supplemental Table 1 - Detailed comorbidities

		A (n=64)	B (n=64)
Depressive disorders	Mild depressive episode (F32.0)	34 (53.1)	36 (56.2)
	Moderate depressive episode (F32.1)	35 (54.7)	33 (51.6)
	Severe depressive episode without psychotic symptoms (F32.2)	30 (46.9)	36 (56.2)
Anxiety disorders	Agoraphobia (F40.0)	32 (50.0)	28 (43.8)
	Social phobias (F40.1)	26 (40.6)	29 (45.3)
	Specific phobias (F40.2)	31 (48.4)	39 (60.9)
	Panic disorder (F41.0)	34 (53.1)	29 (45.3)
	Generalized anxiety disorder (F41.1)	28 (43.8)	36 (56.2)
Adjustment disorders	Acute stress reaction (F43.0)	28 (43.8)	31 (48.4)
	Post-traumatic stress disorder (F43.1)	33 (51.6)	31 (48.4)

Supplemental Table 2 - Psychopathology and family burden

Numbers from Figure 2

Supplemental Table 3 - Kidscreen-52

Detailed scores from Kidscreen-52

- Physical well-being
- Psychological well-being
- Moods and emotions
- Self-perception
- Autonomy
- Parent relation and home life
- Peers and social support
- School environment
- Social acceptance (bullying)
- Financial ressources

Supplemental Table 4 - SAE/SAR/SUSAR

Raw data.

Supplemental Timeline

This timeline will, together with the commits to the version control system, function as a log of the statistical process

- 14.04.2021 - The work on the statistical report with simulated data has been initiated.
- 30.04.2021 - First evaluation of presentation of data using simulated tools.
- 03.05.2021 - Second evaluation of the statistical report.
- 11.05.2021 - Third evaluation of the statistical report.
- 14.05.2021 - Fourth evaluation of the statistical report.
- 19.05.2021 - Fifth evaluation of the statistical report.
- 11.06.2021 - Corrections after sixth evaluation.
- 27.07.2021 - Correction after seventh evaluation.
- 09.03.2022 - Corresponds to the submitted version of the SAP

Supplemental Assumptions

The assumptions for regression models will be assessed using four figures, for each variable.

1. *Residuals vs Fitted*. Used to check the linear relationship assumptions. A horizontal line, without distinct patterns is an indication for a linear relationship, what is good.
2. *Normal Q-Q*. Used to examine whether the residuals are normally distributed. It's good if residuals points follow the straight dashed line.
3. *Scale-Location* (or Spread-Location). Used to check the homogeneity of variance of the residuals (homoscedasticity). Horizontal line with equally spread points is a good indication of homoscedasticity.
4. *Residuals vs Leverage*. Used to identify influential cases, that is extreme values that might influence the regression results when included or excluded from the analysis.

CY-BOCS (primary)

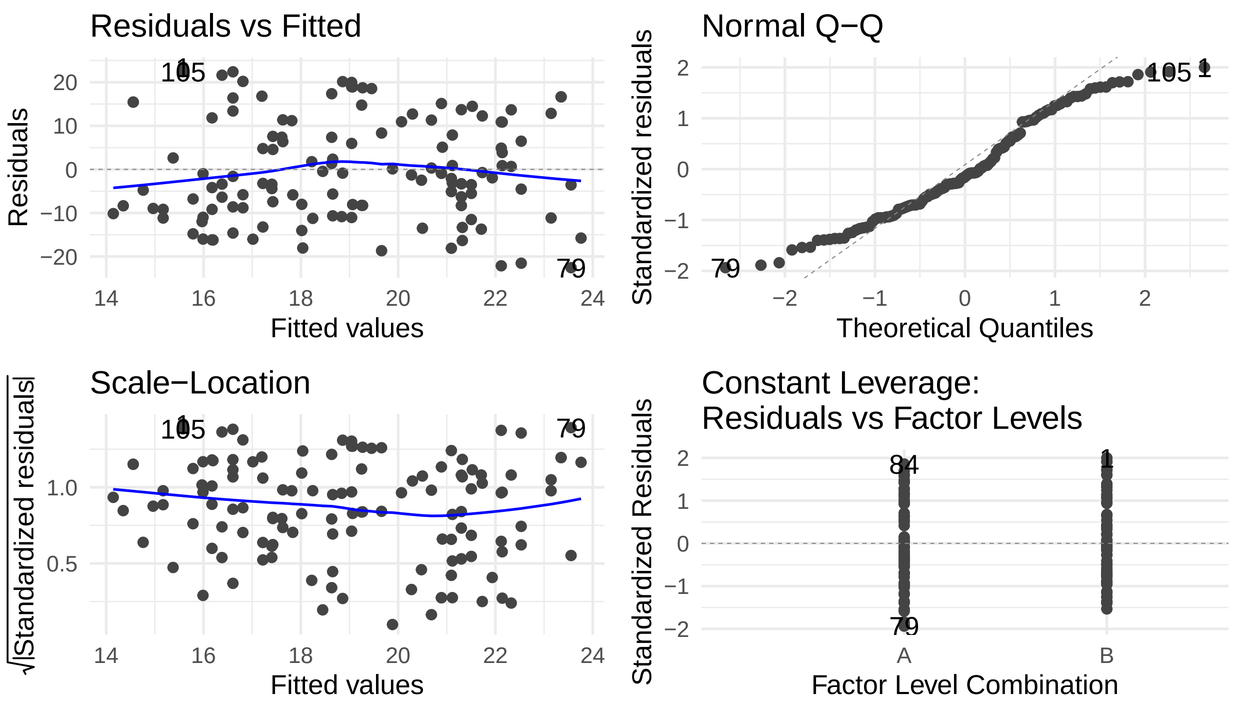

KIDSCREEN-10 (secondary)

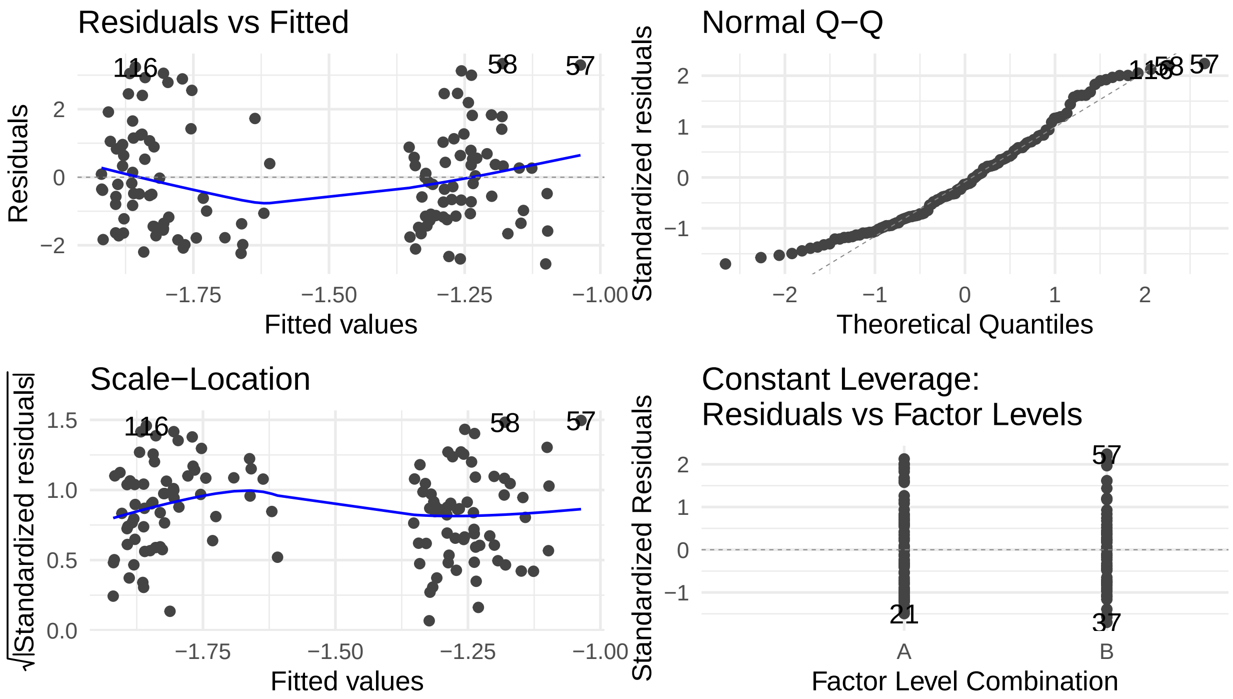

NEQ (secondary)

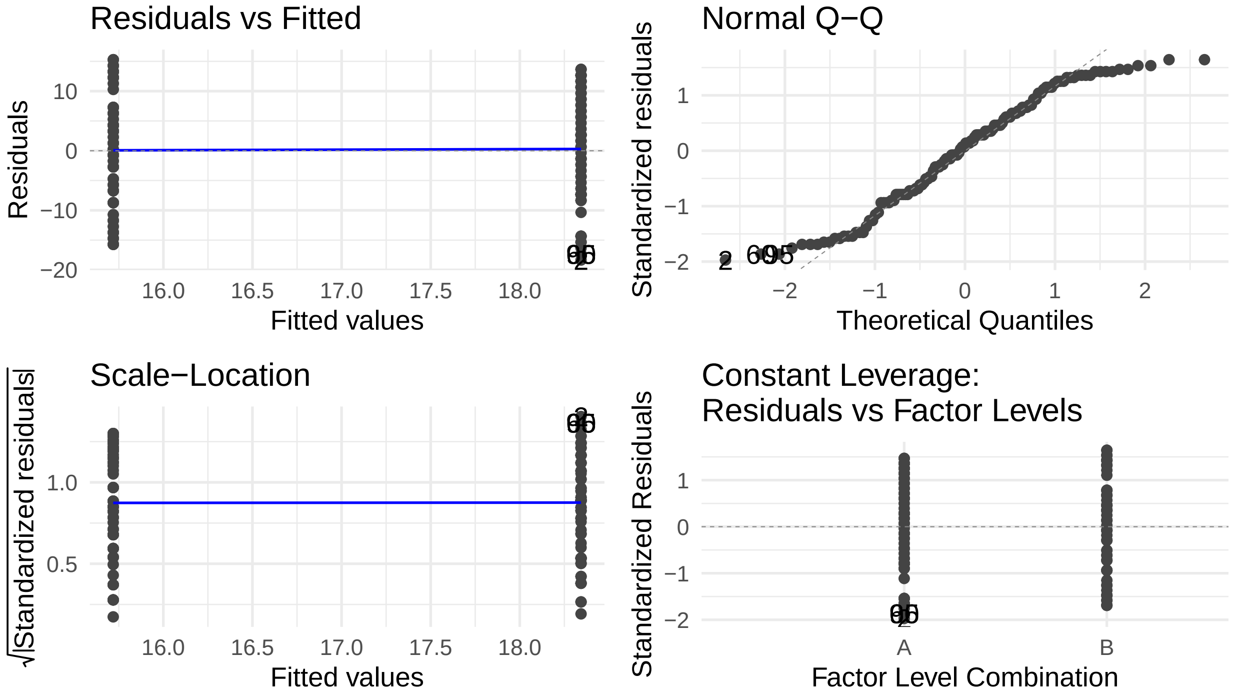

COIS-R (exploratory)

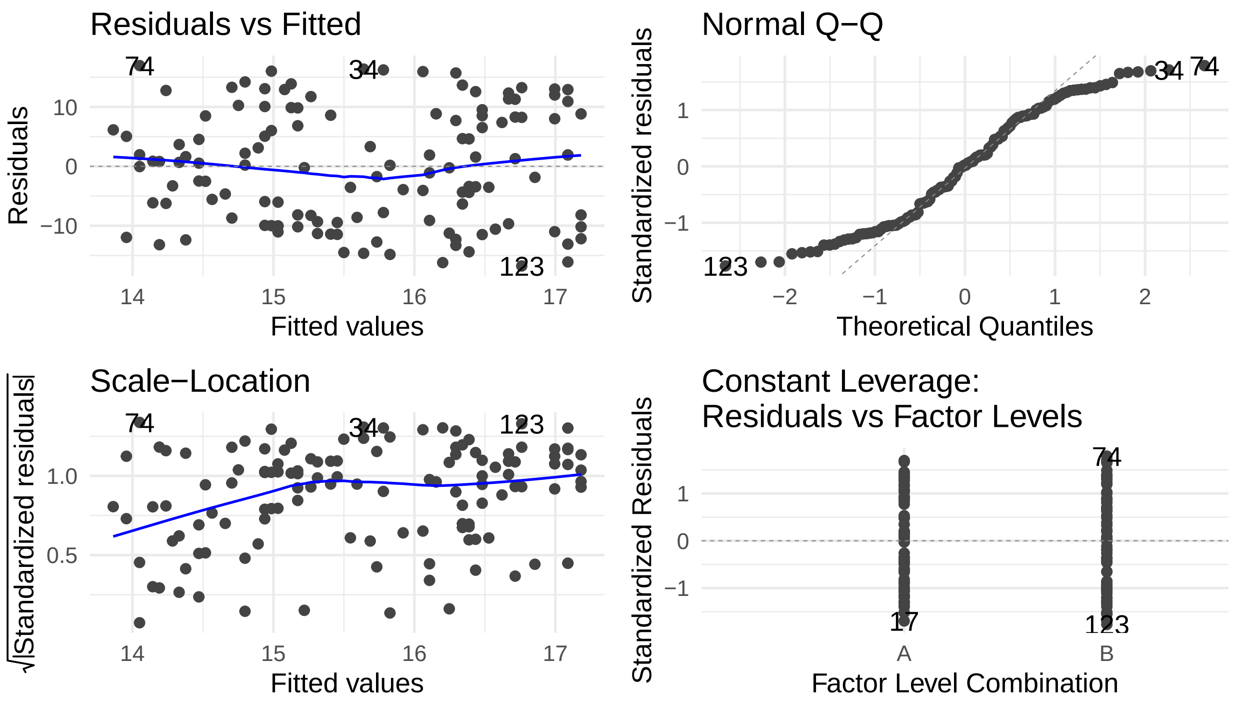

CGI-S (exploratory)

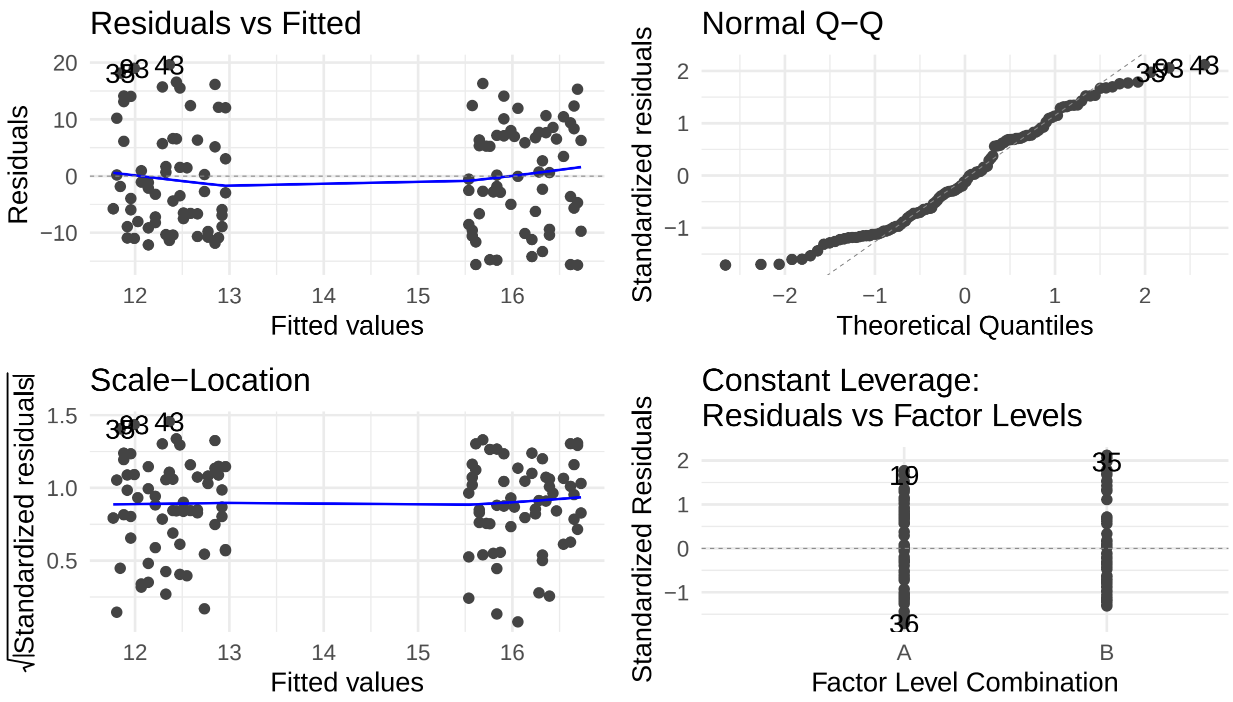

CGI-I (exploratory)

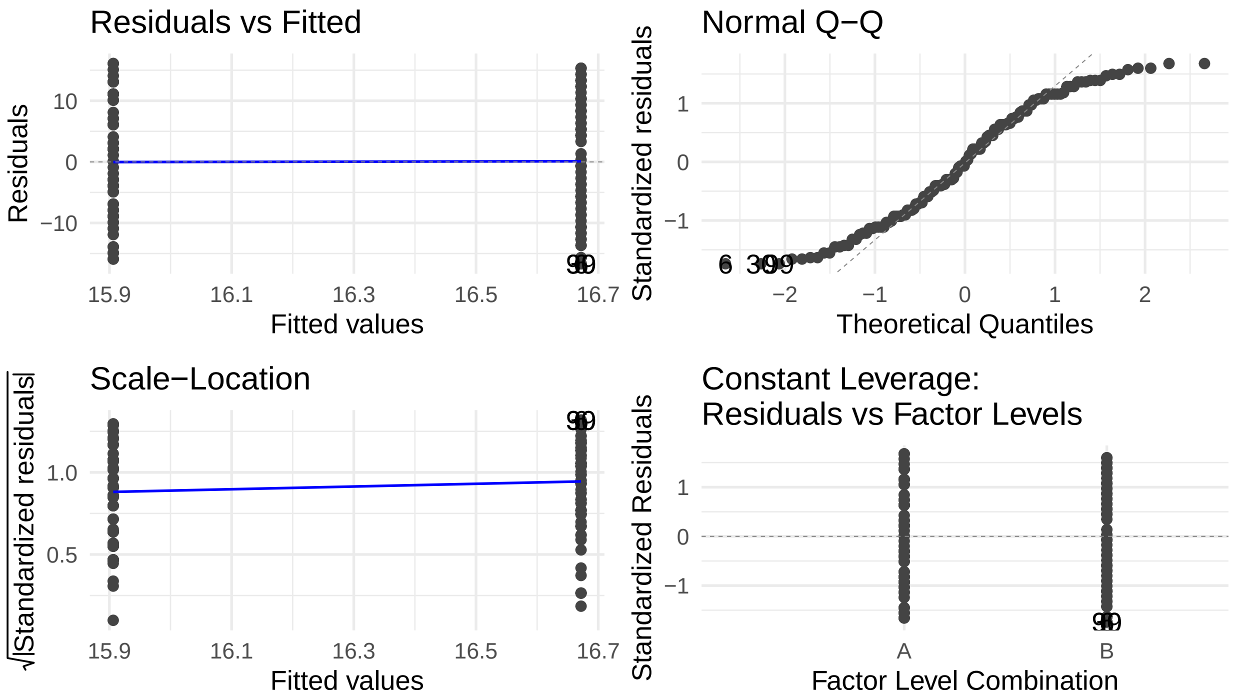

CGAS (exploratory)

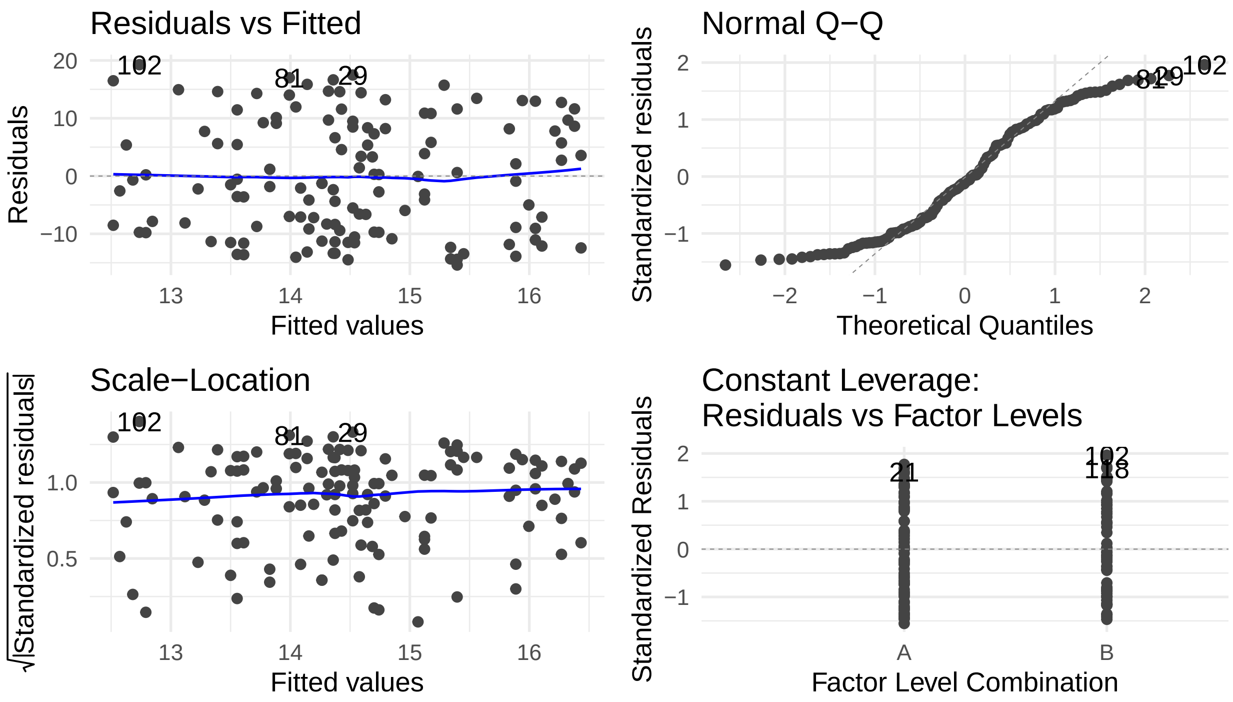

TOCS (exploratory)

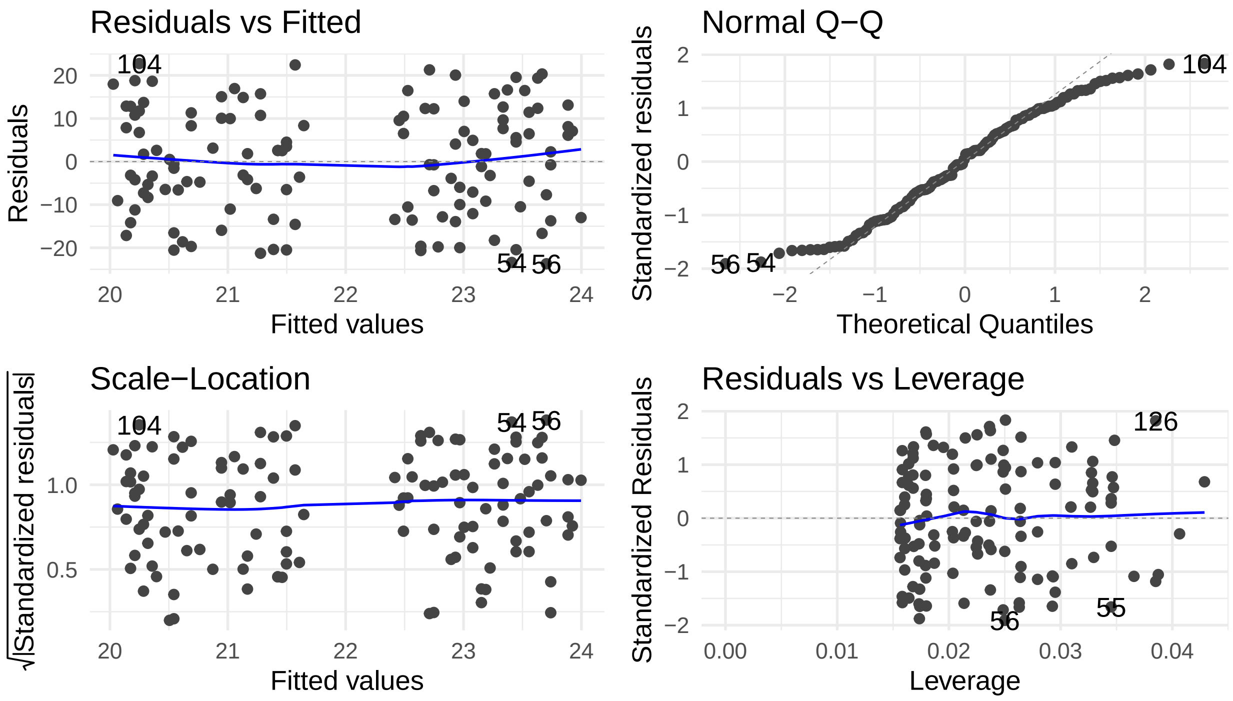

Supplement: Supplementary file 1 — Additional file 1: Table 1. Participant characteristics. Figure 1. CONSORT flow diagram. Figure 2. Psychopathology and family burden. Figure 3. Response status at 16 weeks. Figure 4. Negative effects questionnaire. Supplemental Table 1. Detailed comorbidities. Supplemental Table 2. Psychopathology and family burden. Supplemental Table 3. Kidscreen-52. Supplemental Timeline. Supplemental Assumptions. [file 13063_2022_6799_MOESM1_ESM.pdf]
